# To Facilitate Placing the Dam

**Published:** 1904-06

**Authors:** R. E. Sparks

**Affiliations:** Kingston, Can.


### TO FACILITATE PLACING THE DAM.

By R. E. Sparks, Kingston, Can.

To facilitate the passage of rubber dam between teeth, when the latter are close together: When ready to adjust, smear a little glycerine over the holes, on the side of dam to pass over the teeth. This answers instead of soap, recommended for the purpose, and is much less objectionable to the taste if it should come in contact with the patient's tongue.—*Dominion.*

